# Sja-miR-71a in *Schistosome* egg-derived extracellular vesicles suppresses liver fibrosis caused by schistosomiasis via targeting semaphorin 4D

**DOI:** 10.1080/20013078.2020.1785738

**Published:** 2020-07-09

**Authors:** Lifu Wang, Yao Liao, Ruibing Yang, Zilong Yu, Lichao Zhang, Zifeng Zhu, Xiaoying Wu, Jia Shen, Jiahua Liu, Lian Xu, Zhongdao Wu, Xi Sun

**Affiliations:** a Department of Parasitology of Zhongshan School of Medicine, Sun Yat-sen University, Guangzhou, China; b Key Laboratory of Tropical Disease Control, Ministry of Education, Sun Yat-sen University, Guangzhou, China; c Provincial Engineering Technology Research Center for Biological Vector Control, Guangzhou, China; d Medical Department of Xizang Minzu University, Xianyang, China; e Guangdong Second Provincial General Hospital, Guangzhou, China; f The Third Affiliated Hospital of Sun Yat-sen University, Guangzhou, China; g Nantong University, Nantong, China

**Keywords:** Extracellular vesicles, microRNA, *Schistosoma*-host interactions, liver fibrosis, semaphorin 4D

## Abstract

Schistosomiasis is characterized by liver fibrosis, and studies have indicated that *Schistosoma japonicum* (*S. japonicum*) eggs can limit the progression of liver fibrosis. However, the detailed molecular mechanisms are yet unclear. Extracellular vesicles (EVs) contain a selection of miRNAs for long-distance exchange of information and act as an important pathway for host-parasite communication. This study aimed to explore the potential role of *S. japonicum* egg-derived EVs and its key miRNA in liver fibrosis. Herein, we found that *S. japonicum* egg-derived EVs can inhibit the activation of hepatic stellate cells, which is mediated via the high expression of Sja-miR-71a. Sja-miR-71a in EVs attenuates the pathological progression and liver fibrosis in *S. japonicum* infection. Sja-miR-71a inhibiting TGF-β1/SMAD and interleukin (IL)-13/STAT6 pathways via directly targeting semaphorin 4D (Sema4D). In addition, Sja-miR-71a can also suppress liver fibrosis by regulating Th1/Th2/Th17 and Treg balance. This study contributes to further understanding of the molecular mechanisms underlying *Schistosoma*-host interactions, and Sema4D may be a potential target for schistosomiasis liver fibrosis treatment.

## Introduction

Schistosomiasis is caused by *Schistosoma* species and is a serious parasitic disease throughout the world’s tropical regions. *Schistosoma japonicum* (*S. japonicum*) is widely distributed in the Philippines, Indonesia and the South of China [1]. The female *S. japonicum* worm produces numerous eggs that are transported to the liver. Subsequently, eggs present in the liver elicit host immune responses including granulomatous inflammation and fibrotic reactions. Schistosomiasis is characterized by liver fibrosis and the host’s fibrotic reactions can accelerate the death of the eggs that eventually calcify. Recent studies have shown that *S. japonicum* and *Schistosoma mansoni* (*S. mansoni*) eggs suppress the activation of hepatic stellate cells (HSCs) and lead to the transcriptional downregulation of fibrosis-associated genes [2,3]. Consistently, studies have suggested that soluble antigens from *S. japonicum* eggs can inhibit HSC activation, induce senescence of activated HSCs and facilitate HSCs apoptosis to control the progression of hepatic fibrosis [4–6]. However, the detailed molecular mechanisms remain unclear.

Extracellular vesicles (EVs) contain large quantities of a selection of proteins, lipids and nucleic acids for long-distance exchange of information [7,8]. EVs secreted from various parasites have emerged as important pathways for host-parasite communication. Studies have demonstrated that EVs of parasites can pack specific microRNAs (miRNAs) [9–12], and parasites EVs transfer miRNAs to host and provide parasite–host crosstalk [12,13]. We previously showed that EVs derived from adult *S. japonicum* worms mediate M1 type immune-activity of macrophages [14]. Subsequently, several studies have shown that EVs derived from adult *S. japonicum* and *S. mansoni* worms may play important roles in *Schistosoma*–host interactions [15–19]. Zhu et al. showed release of EVs containing small RNAs from the eggs of *S. japonicum* [20]. Meningher et al. showed that *S. mansoni* worms derived EVs enclosed miRNAs modulate host T helper cell differentiation [21]. Among the various active components within EVs, miRNAs are regarded as essential to the function of EVs [22,23].

miRNAs are small non-coding RNAs that negatively regulate gene expression at the post-transcriptional or translational level via complementary binding to the 3′-untranslated region (UTR) [24–26]. Recent studies have shown that host miRNAs play a critical role in liver fibrosis in *S. japonicum* infection [27–29]. However, few studies have yet analysed the roles of miRNAs from *S. japonicum* in liver fibrosis.

Therefore, we aimed to analyse the role of *S. japonicum* egg-derived EVs and its key miRNA in liver fibrosis to further understand the molecular mechanisms underlying *Schistosoma*–host interactions. Interestingly, in this study, we showed that *S. japonicum* eggs secreted EVs that can inhibit liver fibrosis in *S. japonicum* infection via high expression of Sja-miR-71a.

## Materials and methods

### Animals and ethics

Male BALB/c mice were purchased from Guangdong Medical Laboratory Animal Centre. To induce infection, mice were exposed percutaneously to *S. japonicum* cercariae that were shed from lab-infected snails (*Oncomelania hupensis*), obtained from the National Institute of Parasitic Disease, Chinese Centre for Disease Control and Prevention. All animal experiments were approved by the Sun Yat-sen University Committee for Animal Research and conformed to the Guidelines for the Care and Use of Laboratory Animals of the National Institute of Health in China.

### Patients and serum

All human serum came from patients infected with *S. japonicum* and healthy controls in Nanchang, Jiangxi, China. All human serum was obtained with informed consent and approved by the Ethics Committee of Zhongshan School of Medicine, Sun Yat-sen University (2019 no. 54).

### EVs purification and identification

To prepare *S. japonicum* EVs, *S. japonicum* eggs and worms were collected from infected mice at 45 days post-infection. EVs in eggs were analysed by transmission electron microscopy. EVs were purified by differential centrifugation and following validation by electron microscopy and Nanoparticle Tracking Analyses (NTA). For additional details, see supplementary information.

### In vivo imaging system

*S. japonicum* egg-derived EVs and *S. japonicum* adult-derived EVs were stained with DiR (1,1′‐dioctadecyl‐3,3,3′,3′‐tetramethylindotricarbocyanine iodide). Mice were injected via the tail vein with DiR-labelled EVs. Anaesthetized mice were placed into the In-Vivo Imaging System (Maestro, USA) at 2, 4, 6, 8, 24, and 48 h after EVs injection. At the end of the last total body scan 48 h post-injection, mice were euthanized, and organs were harvested and placed into the In-Vivo Imaging System for *ex vivo* imaging.

### EVs uptake experiment

The human liver stellate cell line (LX2) (Sure Biological Technology, China) was incubated with PKH26-labelled *S. japonicum* egg-derived EVs for 1 h, and a confocal analysis was carried out to evaluate EVs internalization. DAPI was used to detect nuclei, Alexa Fluor Phalloidin-FITC was used to label actin.

### Cell culture and treatment

For EVs and Recombinant Human Semaphorin 4D (Sema4D) (Novoprotein, China) treatment, LX2 cells were exposed to recombinant TGF-β1 (PeproTech, USA) or EVs. For miRNA mimic and siRNA treatment, cells were incubated in serum-free medium for starvation overnight. Cells were then stimulated with miRNA mimic and siRNA using RNAiMAX (Invitrogen, USA). For additional details, see supplementary information.

### RNA extraction and quantitative reverse transcription PCR (qRT-PCR)

Total RNA was harvested using TRIzol according to manufacturer’s instructions. The expression of target mRNA and miRNA was determined using the SYBR Green Master Mix kit (Takara, Japan). GAPDH, β-actin or U6 snRNA was used as an internal control, and the fold change was calculated by the 2^−ΔΔCT^ method. For additional details, see supplementary information.

### Small RNA sequencing and analysis

Total RNA was isolated from EVs and subjected to quantitative and qualitative analyses to ensure the use of qualified samples for sequencing and analysis. For additional details, see supplementary information.

### Western blotting

Liver tissues and LX2 cells were homogenized with RIPA lysis buffer in the presence of freshly added protease and phosphatase inhibitors (Thermo Fisher Scientific, USA). Lysates were subjected to 10% sodium dodecyl-polyacrylamide gel electrophoresis and transferred to a polyvinylidene fluoride blotting membrane. The membranes were then immunoblotted with antibodies. For additional details, see supplementary information.

### Histopathology and fibrosis measurement

Liver tissues from the infected mice were weighed and digested overnight in 4% potassium hydroxide, released eggs were counted under a dissecting microscope. The index of liver and spleen was calculated based on the following formula: total weight of mouse liver or spleen (mg)/total weight of mouse body (g). The area of egg granulomas was measured based on H&E-stained sections by using the Image-Pro Plus 6.0 software. The area of each granuloma in section was measured. Fibrosis was observed using liver sections stained with Masson’s trichrome staining, and the integrated optical density (IOD) was analysed by Image-Pro Plus 6.0. Serum alanine aminotransferase (ALT) levels were detected by KingMed Diagnostics (Guangzhou, China).

### Recombinant adeno-associated virus (rAAV) vectors and transduction

HBAAV2/9-Sja-miR-71a-GFP and HBAAV2/9-GFP viral particles were purchased from Hanbio Biotechnology Co., Ltd. LX2 cells were transfected with rAAV (2 × 10^9^ genome copies/200 µl). Ninety-six hours after transduction Sja-miR-71a levels were quantified by qRT-PCR. For mice transduction, mice were injected via the tail vein with rAAV (1.5 × 10^11^ v.g./mouse) 10 days after infection with *S. japonicum*. The green fluorescent protein (GFP) expression of rAAV in mice was observed using *in vivo* imaging system and fluorescence microscopy.

### Immunohistochemistry and immunofluorescence analysis

Liver tissues were fixed in 4% neutral-buffered formalin and embedded in paraffin. Sections were dewaxed and incubated with GFAP, α-SMA, Sema4D, Plexin B1, CD72, Plexin B2, IL-13Rα1, p-JKA1, p-STAT6, TGF-β1, p-SMAD2/3, SMAD4, CD3e and CD4 antibodies overnight at 4°C. The sections were then incubated with the indicated secondary antibodies. The sum of the IOD was analysed by Image-Pro Plus 6.0. For additional details, see supplementary information.

### Flow cytometry

For spleens, single-cell suspensions were prepared. Cells were stained with CD3e, CD4 and CD25; the cells were then stained with Foxp3 and T-bet antibodies after fixation and permeabilization. For Th1, Th2 and Th17 subsets, cells were washed and stained for CD3e and CD4. In addition, cells were stained with intracellular interferon-γ (IFN-γ), IL-4 and IL-17A antibodies. For additional details, see supplementary information.

### Dual-luciferase reporter assay

Cells were transfected with the Sema4D UTR reporter plasmid together with Sja-miR-71a mimic or control mimic for 48 h using RNAiMAX (Invitrogen, USA). The Luciferase Assay Reagent (Beyotime Biotechnology, China) was added to the cells, and luciferase activities were detected using the Infinite F500 Multimarker Analyser (TECAN, Austria) according to the manufacturer’s instructions. Luciferase activity was normalized to *Renilla* luciferase activity.

### Statistical analysis

Results are expressed as mean ± SD values. The data of both groups were analysed using unpaired two-sample *t*-test. Multiple comparisons between more than two groups were analysed by one-way ANOVA test or Kruskal–Wallis test (non-parametric). The value of *P* < 0.05 was considered statistically significant.

## Results

### *S. japonicum* egg-derived EVs inhibit the activation of HSCs in vitro

**Figure 1.**
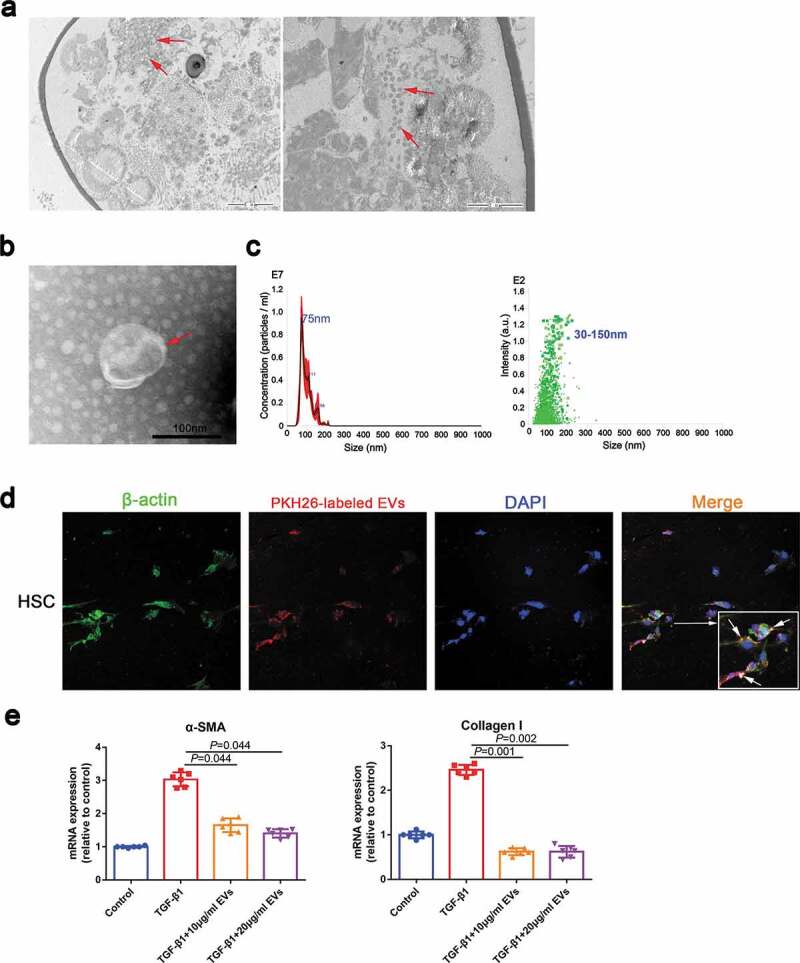
*S. japonicum* egg-derived EVs inhibit the activation of HSCs. (a). EVs in *S. japonicum* eggs were analysed by transmission electron microscopy (TEM). (b). *S. japonicum* eggs-derived EVs were purified and analysed by negative-staining TEM. (c). *S. japonicum* egg-derived EVs particles were investigated by Nanoparticle Tracking Analyses (NTA). (d). HSCs (LX2) were incubated with PKH26-labelled *S. japonicum* egg-derived EVs, and laser scanning confocal microscopy was carried out to evaluate EVs internalization. (e). HSCs (LX2) were treated with *S. japonicum* egg-derived EVs (10 μg/mL and 20 μg/mL) for 48 h. α-SMA and collagen I expressions were analysed by quantitative reverse transcription PCR (qRT-PCR). Results are shown as mean ± SD (Kruskal–Wallis test).

To test whether *S. japonicum* eggs release EVs, we first conducted electron microscopy studies and identified the presence of EVs in *S. japonicum* eggs (Figure 1(a), arrows). Next, EVs were isolated from the conditioned media of *S. japonicum* eggs. Negative-staining TEM analysis of EVs revealed a typical size of 50–150 nm with a characteristic cup-shaped morphology (Figure 1(b), arrows). Further, the size distribution profile of the EVs was investigated by NTA, which revealed a peak size of 75 nm (Figure 1(c)).

To determine the functional effects of *S. japonicum* egg-derived EVs on *S. japonicum*–host interactions, we evaluated whether *S. japonicum* egg-derived EVs can reach the host organs to perform their functions. Our data showed that *S. japonicum* egg-derived EVs can reach host organs including the heart, brain, liver, spleen, kidney, lung and thymus (Supplemental Figure 1(a, b)). We hypothesized that the eggs located in the centre of the granuloma secrete EVs enter the cytosol of HSCs and regulate activation of HSCs and thereby regulate hepatic fibrosis. As shown in Figure 1(d), *S. japonicum* egg-derived EVs can be internalized by HSC and reduced the α-SMA and Collagen I expression of TGF-β1-treated human LX2 cells (Figure 1(e)), suggesting that *S. japonicum* egg-derived EVs exert an antifibrotic effect.

### Sja-miR-71a is highly expressed in *S. japonicum* egg-derived EVs and can inhibit the activation of HSCs

To identify the components responsible for the inhibit HSCs activation effect of *S. japonicum* egg-derived EVs, we assessed the expression profiles of miRNA in the *S. japonicum* egg-derived EVs. We compared the differences of miRNA expression profiles in *S. japonicum* egg-derived EVs and *S. japonicum* adult-derived EVs (Supplementary Figure 2(a)). Among the miRNAs of *S. japonicum* egg-derived EVs, Sja-miR-71a was the most highly expressed, and its expression was 270 times greater than that in *S. japonicum* adult EVs (Supplementary Figure 2(a), Supplementary Table 1).

**Figure 2.**
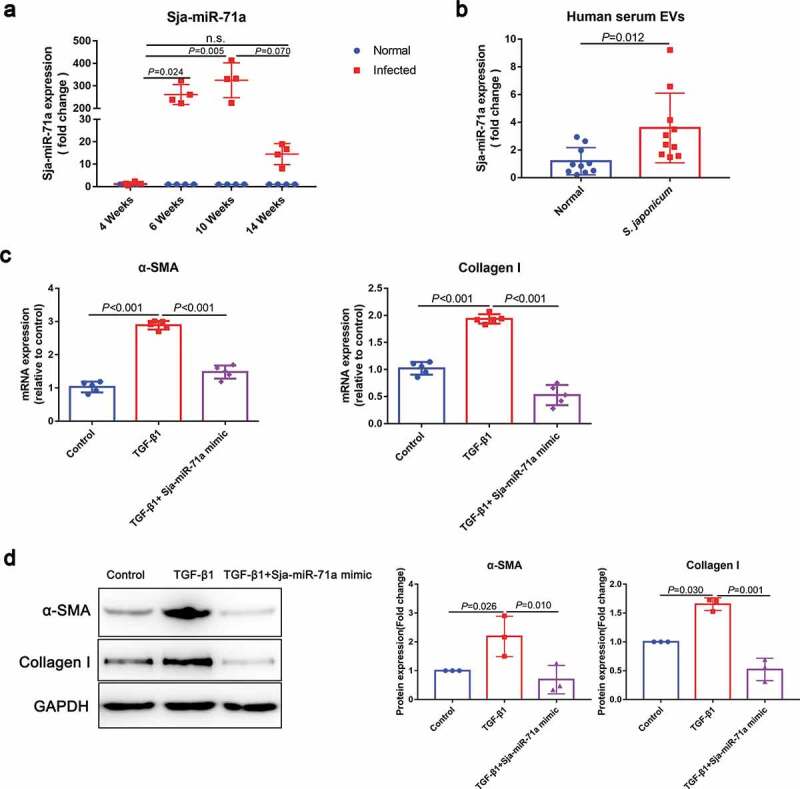
*S. japonicum* egg-derived EVs inhibit HSCs activation via high expression of Sja-miR-71a. (a). Expression levels of Sja-miR-71a in mice livers in different stages of *S. japonicum* infection were analysed by qRT-PCR (n = 4 per group). (b). Sja-miR-71a expression in serum EVs of patients with schistosomiasis japonicum was analysed by qRT-PCR (*n* = 9–10 per group). (c, d). HSCs (LX2) were treated with PBS, TGF-β1 (10 ng/mL), and TGF-β1 (10 ng/mL) + Sja-miR-71a (50 nM) for 72 h. α-SMA and Collagen I expression were analysed by qRT-PCR and western blotting. Results are shown as mean ± SD. (a): Kruskal–Wallis test; (b): Unpaired two-sample *t*-test; (c and d): One-way ANOVA.

Interestingly, elevated Sja-miR-71a expression in mice total liver tissues was also observed from sixth week (*S. japonicum* spawn in large numbers) after infection (Figure 2(a)). Both *S. japonicum* egg-derived EVs and *S. japonicum* adult-derived EVs can reach host liver, while *S. japonicum* egg-derived EVs carried more Sja-miR-71a to the liver (Supplemental Figure 1, Supplemental Figure 2(b)). We then determined Sja-miR-71a expression in serum EVs of patients with schistosomiasis japonicum. Interestingly, Sja-miR-71a expression can be detected in serum EVs of patients (Figure 2(b)), suggesting that Sja-miR-71a is carried by *S. japonicum* egg-derived EVs for long-distance exchange of information. We further explore the potential role of Sja-miR-71a in the activation of HSCs. We found significantly decreased expression of α-SMA and Collagen I in TGF-β1-treated LX2 cells after treatment with Sja-miR-71a (Figure 2(c,d)). Collectively, our results suggest that the inhibitory effect of *S. japonicum* egg-derived EVs on the activation of HSCs is mediated by the high expression of Sja-miR-71a.

### Sja-miR-71a in *S. japonicum* egg-derived EVs attenuates the pathological progression and liver fibrosis in *S. japonicum* infection

**Figure 3.**
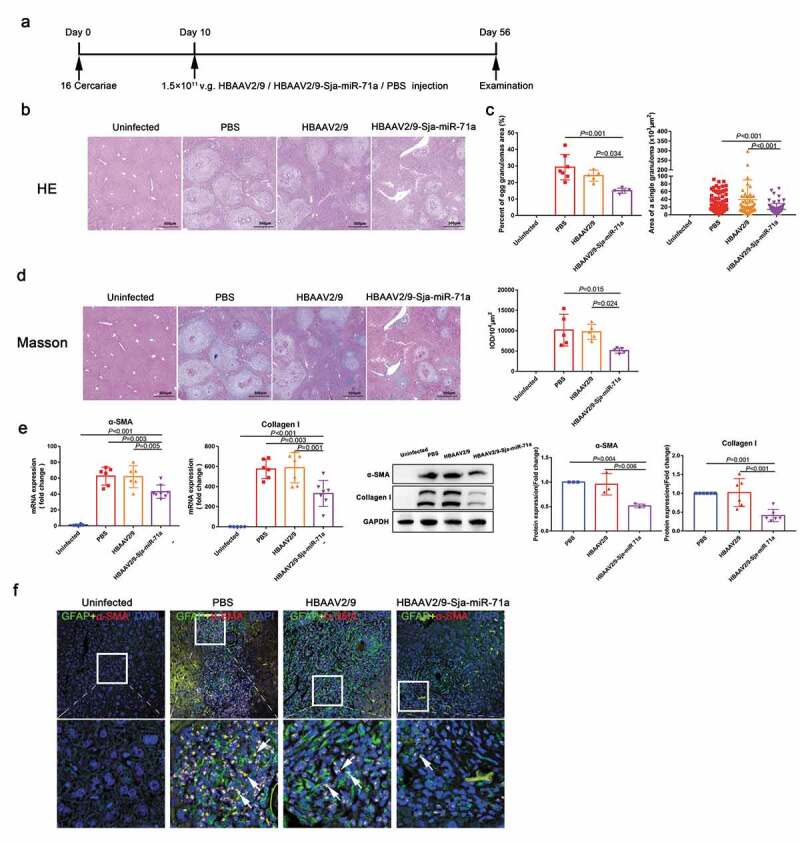
Sja-miR-71a in *S. japonicum* egg-derived EVs attenuates the pathological progression of liver fibrosis in *S. japonicum* infection. (a). Time schedule for parasite infection and intravenous injections of rAAV vectors or PBS and sample examination. (b). H&E staining of liver sections. (c). The percentage of granulomatous area and the area of a single granuloma was measured from H&E sections using Image-Pro Plus 6.0 software (*n* = 5–7 per group). (d). Fibrosis was observed using liver sections stained with Masson’s trichrome staining, and integrated optical density (IOD) was analysed by Image-Pro Plus 6.0 (*n* = 5 per group). (e). Expression levels of α-SMA and collagen I in mice livers were analysed by qRT-PCR and western blotting (*n* = 5–8 per group). (f). The co-localization of GFAP (glial fibrillary acidic protein) and α-SMA in liver sections was observed, DAPI was used as a counterstain. Results are shown as mean ± SD (one-way ANOVA Dunnett’s multiple comparison test).

To investigate if Sja-miR-71a in *S. japonicum* egg-derived EVs was involved in the progression of hepatic schistosomiasis, HBAAV2/9-Sja-miR-71a was successful constructed (Supplementary Figure 3(a)). Mice were infected with *S. japonicum* and administered with the HBAAV2/9- Sja-miR-71a (Figure 3(a)). *In vivo* imaging system confirmed that HBAAV2/9-Sja-miR-71a could colonize the liver, spleen, thymus, and colon (Supplementary Figure 3(b)). In addition, liver sections and qRT-PCR reflected the highly hepatic tropism of HBAAV2/9-Sja-miR-71a (Supplementary Figure 3(c, d)). We observed that hepatosplenomegaly was markedly reduced after HBAAV2/9-Sja-miR-71a transfection, and the liver and spleen indices significantly reduced after HBAAV2/9-Sja-miR-71a treatment (Supplementary Figure 4(a, b)).

Interestingly, H&E staining of liver sections showed that compared with those in the control group, egg granulomas, per cent of egg granuloma areas, and area of a single granuloma in the HBAAV2/9-Sja-miR-71a group were significantly shrunk (Figure 3(b,c)). There were no significant differences with respect to egg burden between these groups, indicating the Sja-miR-71a treatment had no effect on parasite reproduction (Supplementary Figure 5(a)). Furthermore, Masson’s trichrome staining of liver sections showed significantly reduced collagen deposition in the HBAAV2/9-Sja-miR-71a-treated mice (Figure 3(d)). In addition, mice treated with HBAAV2/9-Sja-miR-71a showed significantly lower circulating levels of ALT than those treated with HBAAV2/9 alone, suggesting the Sja-miR-71a alleviated hepatocellular damage (Supplementary Figure 5(b)). Additionally, we analysed the expression of α-SMA and Collagen I in the livers of infected mice. As expected, significantly reduced expression of α-SMA and Collagen I was observed in the HBAAV2/9-Sja-miR-71a-treated mice (Figure 3(e)). To further investigate the activation of HSCs in livers, we performed immunofluorescent dual staining. α-SMA co-localised with HSCs marker (glial fibrillary acidic protein, GFAP) revealed that α-SMA in HSCs was significantly reduced after treated with HBAAV2/9-Sja-miR-71a (Figure 3(f), arrows). Together, our findings show that Sja-miR-71a attenuates the pathological progression and liver fibrosis in *S. japonicum* infection.

### Sema4D is a direct target of Sja-miR-71a

**Figure 4.**
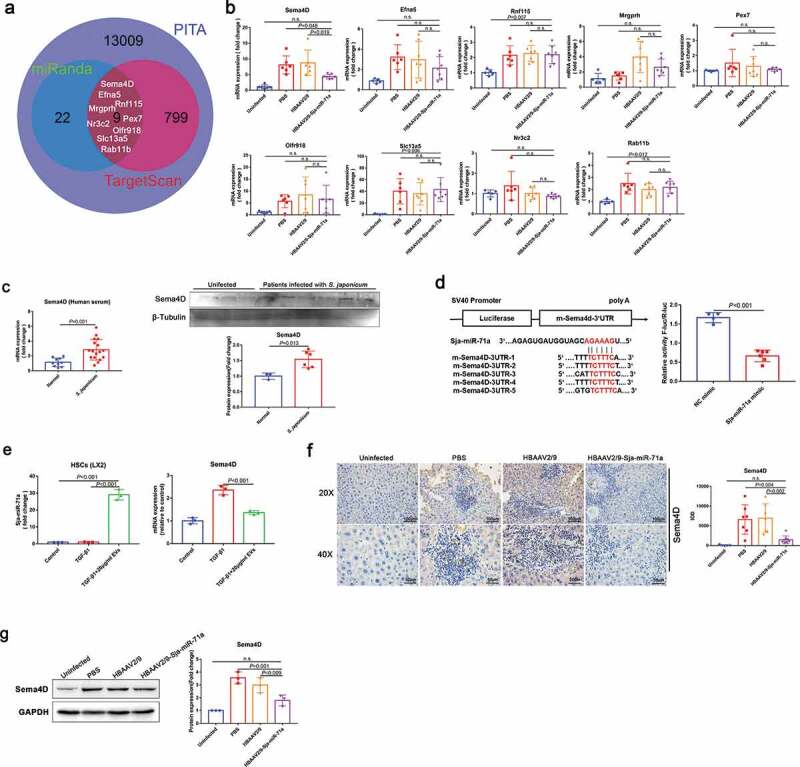
Sema4D is a direct target of Sja-miR-71a. (a). Three databases (miRanda, PITA, and TargetScan) were used to identify potential Sja-miR-71a targets. (b). mRNA expression of nine exemplary predicted targets of Sja-miR-71a in mice livers was analysed by qRT-PCR (*n* = 5–7 per group). (c). Sema4D in serum of schistosomiasis japonicum patients were analysed by qRT-PCR (*n* = 10–17 per group) and western blotting (*n* = 3–6 per group). (d). The wild-type m-Sema4D-3′- untranslated region (UTR) was cloned into psi-CHECK-2, and five predicted binding sites of Sja-miR-71a in the 3′-UTR of Sema4D gene; Dual-luciferase reporter assay performed on 293 T cells transfected with Sema4D UTR reporter plasmid together with Sja-miR-71a mimic or control mimic. (e). HSCs (LX2) were treated with *S. japonicum* egg-derived EVs (20 μg/mL) for 24 h. Sja-miR-71a and Sema4D expressions were analysed by qRT-PCR. (f, g). Expression of Sema4D in mice livers was analysed by immunohistochemistry (the sum of the IOD was analysed by Image-Pro Plus 6.0, *n* = 5–8 per group) and western blotting. Results are shown as mean ± SD. (b, e, f and g): One-way ANOVA Dunnett’s multiple comparison test. (c and d): Unpaired two-sample *t*-test.

To further evaluate the molecular mechanisms of Sja-miR-71a in liver fibrosis, we aimed to identify direct target genes that might be controlled by Sja-miR-71a expression. Three databases (miRanda, PITA, and TargetScan) were used to identify potential Sja-miR-71a targets. Venn diagram showing there are nine potential target genes overlap in three database predictions (Figure 4(a)). Among them, only Sema4D was significantly down-regulated after treated with HBAAV2/9-Sja-miR-71a (Figure 4(b)). In addition, we found Sema4D in serum of schistosomiasis japonicum patients were increased than healthy controls (Figure 4(c)). Furthermore, as shown in Figure 4(d), Sja-miR-71a had five binding sites in the 3′-UTR of Sema4D. Thus, based on the analysis above, we selected the Sema4D as a potential target gene for further investigation.

To verify whether Sema4D is a direct target of Sja-miR-71a, we generated a luciferase reporter plasmid containing the 3′-UTR of Sema4D flanking the putative Sja-miR-71a binding sites. The dual-luciferase reporter assay revealed that compared to NC mimic-transfected cells, Sja-miR-71a significantly reduced the luciferase activity of the Sema4D construct (Figure 4(d)). We found *S. japonicum* egg-derived EVs carried Sja-miR-71a to the HSCs, and TGF-β-mediated HSCs highly expressed Sema4D can be downregulated by *S. japonicum* egg-derived EVs (Figure 4(e)). To further determine Sema4D is regulated by Sja-miR-71a, we analysed the Sema4D protein expression level in mice livers. As expected, Sema4D protein was markedly down-regulated in mice livers treated with HBAAV2/9-Sja-miR-71a than control groups (Figure 4(f,g)). Next, we examined the expression of Plexin B1 and CD72, as Sema4D binds with both these receptors [30]. As proven by the immunohistochemistry and western blotting results, the expression of both Plexin B1 and CD72 was lower in the HBAAV2/9-Sja-miR-71a-treated than the *S. japonicum*-infection control groups (Supplementary Figure 6(a, b)). Taken together, these experiments showed that Sema4D was markedly increased after infection with *S. japonicum*, and Sja-miR-71a could inhibit Sema4D expression by directly interacting with its 3′-UTR.

### Sja-miR-71a inhibits HSCs activation to alleviate liver fibrosis by inhibiting Sema4D

**Figure 5.**
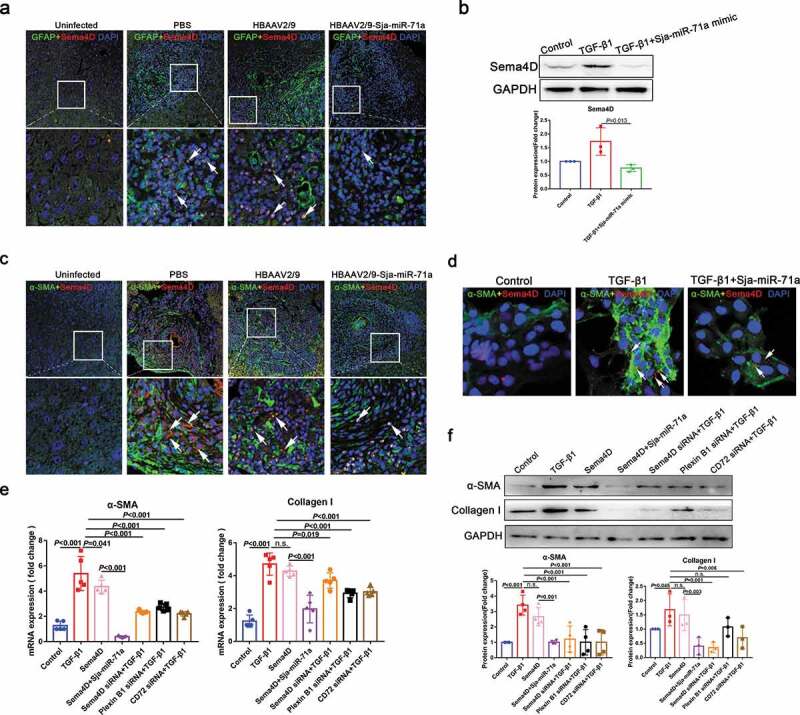
Sja-miR-71a inhibits HSCs activation to alleviate liver fibrosis by inhibiting Sema4D. (a). The co-localization of GFAP and Sema4D in liver sections was observed, DAPI was used as a counterstain. (b). Expression level of Sema4D was determined by western blotting for LX2 cells treated with PBS, TGF-β1 and TGF-β1+ Sja-miR-71a mimic. (c). The co-localization of α-SMA and Sema4D in liver sections was observed, DAPI was used as a counterstain. (d). The co-localization of α-SMA and Sema4D in LX2 cells (treated with PBS, TGF-β1 and TGF-β1+ Sja-miR-71a mimic) was observed, DAPI was used as a counterstain. (e, f). LX2 cells treated with PBS, TGF-β1, Recombinant Human Sema4D, Recombinant Human Sema4D+Sja-miR-71a mimic, Recombinant Human Sema4D+TGF-β1, Human Plexin B1 siRNA+TGF-β1 and Human CD72 siRNA+TGF-β1 for 48 h. Expression levels of α-SMA and Collagen I were determined by qRT-PCR (*n* = 5 per group) and western blotting (*n* = 4 per group). Results are shown as mean ± SD. One-way ANOVA test was used for statistical analysis.

Next, we tested whether Sja-miR-71a inhibits HSCs activation to alleviate liver fibrosis by inhibiting Sema4D. Sema4D co-localised with HSCs marker (GFAP) revealed that Sema4D in HSCs was significantly increased in mice livers after infection with *S. japonicum* and could be reduced by treated with HBAAV2/9-Sja-miR-71a (Figure 5(a), arrows). In addition, we demonstrated that TGF-β-mediated LX2 cells highly expressed Sema4D, Plexin B1 and CD72 can be downregulated by Sja-miR-71a (Figure 5(b), Supplementary Figure 6(c)). Interestingly, Sema4D co-localised with α-SMA revealed that the change of α-SMA expression in livers was consistent with the change of Sema4D, α-SMA and Sema4D were significantly reduced after treated with HBAAV2/9-Sja-miR-71a (Figure 5(c), arrows). We further demonstrated that highly expressed α-SMA in TGF-β-mediated LX2 cells can be downregulated after Sja-miR-71a mimic inhibit Sema4D (Figure 5(d)). We then assessed whether Sema4D treatment would activate HSCs. As shown in Figure 5(e,f), α-SMA and Collagen I were significantly upregulated in LX2 cells after treatment with Recombinant Human Sema4D, and Sja-miR-71a could inhibit Sema4D-induced α-SMA and Collagen I expression. Furthermore, we assessed whether down-regulation of Sema4D and Sema4D receptors in HSCs would inhibit TGF-β-mediated HSCs activation. We used siRNA to knockdown *Sema4D, Plexin B1*, and *CD72* genes in LX2 cells. The results showed levels of α-SMA and collagen were decreased after knockdown *Sema4D, Plexin B1*, and *CD72* (Figure 5(e,f)). Our findings show that Sja-miR-71a inhibits HSCs activation to alleviate liver fibrosis by inhibiting Sema4D.

### Sja-miR-71a suppresses liver fibrosis via the Sema4D/TGF-β1 axis and Sema4D/IL-13 axis

**Figure 6.**
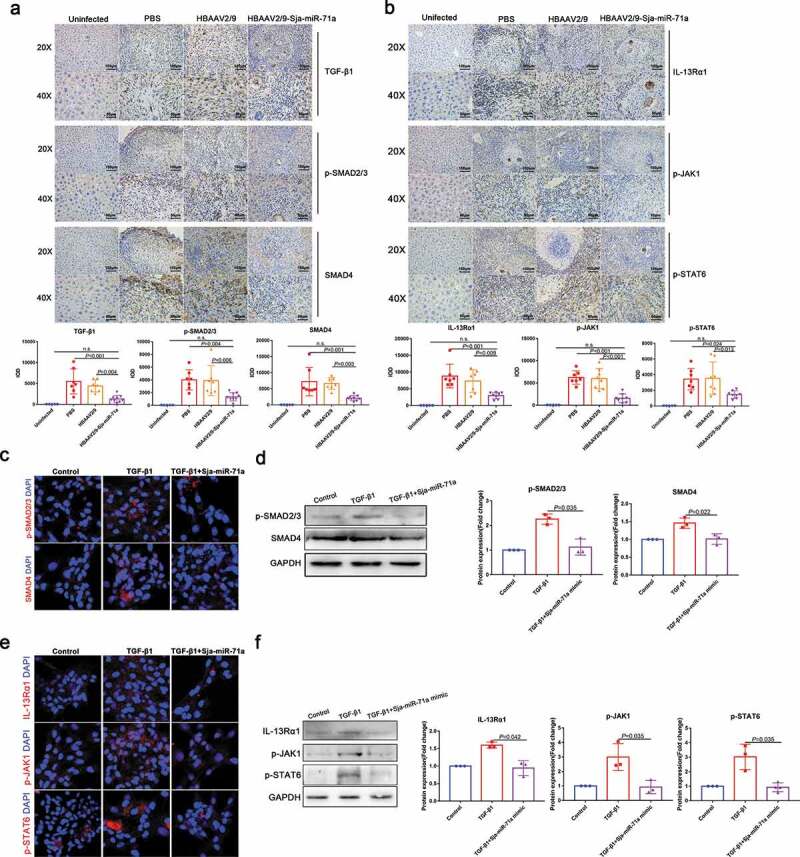
Sja-miR-71a suppresses liver fibrosis via Sema4D/TGF-β1 and Sema4D/IL-13 axes. (a). Immunohistochemical analysis of TGF-β1, phosphorylated SMAD2/3 (p-SMAD2/3) and SMAD4 in mice livers, and the sum of the IOD was analysed by Image-Pro Plus 6.0 (*n* = 5–9 per group). (b). Expression of IL-13Rα1, phosphorylated JAK1 (p-JAK1) and phosphorylated SATA6 (p-SATA6) in mice livers were analysed by immunohistochemistry, and the sum of the IOD was analysed by Image-Pro Plus 6.0 (*n* = 5–9 per group). (c, d). HSCs (LX2) were treated with PBS, TGF-β1 (10 ng/mL) and TGF-β1 (10 ng/mL) +Sja-miR-71a (50 nM) for 72 h; p-SMAD 2/3 and SMAD4 were analysed by immunofluorescence analysis and western blotting. (e, f). HSCs (LX2) were treated with PBS, TGF-β1 (10 ng/mL) and TGF-β1 (10 ng/mL)+Sja-miR-71a (50 nM) for 72 h; IL-13Rα1, p-JAK1 and p-SATA6 were analysed by immunofluorescence analysis and western blotting. Results are shown as mean ± SD. One-way ANOVA test was used for statistical analysis.

Sema4D can directly increase TGF-β1 expression, and IL-13 and TGF-β1 expression was decreased in a Sema4D ^−/−^ mouse model of experimental asthma [31,32]. In addition, both TGF-β1/SMAD and IL-13/STAT6 pathways have been identified as major pathways that promote liver fibrosis in schistosomiasis by activating HSCs [33,34]. These findings led us to speculate that Sja-miR-71a-induced suppression of liver fibrosis is regulated by the Sema4D/TGF-β1 and Sema4D/IL-13 axes. To test our hypothesis, TGF-β1/SMAD and IL-13/STAT6 pathways were identified. We showed that the expression of TGF-β1 was up-regulated in the schistosome-infected livers and down-regulated after HBAAV2/9-Sja-miR-71a treatment (Figure 6(a)). Moreover, phosphorylated SMAD2/3 (p-SMAD 2/3) and SMAD4 – proteins that are pivotal downstream effectors of TGF-β1 and convey signals from TGF-β receptors to the nucleus – were up-regulated in the schistosome-infected livers and down-regulated after HBAAV2/9-Sja-miR-71a treatment (Figure 6(a)). Furthermore, we observed that IL-13Rα1 and IL-13 receptor were down-regulated in HBAAV2/9-Sja-miR-71a-treated schistosome-infected livers compared to schistosome-infection control groups (Figure 6(b)). Phosphorylated JAK1 (p-JAK1) and phosphorylated SATA6 (p-SATA6), two key molecules in the IL-13/STAT6 pathway were highly expressed in the schistosome-infected livers and down-regulated by HBAAV2/9-Sja-miR-71a treatment (Figure 6(b)).

Next, we verified whether Sja-miR-71a suppresses liver fibrosis through the Sema4D/TGF-β1 axis and Sema4D/IL-13 axis in HSCs. We transfected LX2 cells with Sja-miR-71a mimic *in vitro*. Interestingly, transfection with Sja-miR-71a mimic decreased the expression of TGF-β-mediated p-SMAD 2/3 and SMAD4 (Figure 6(c,d)). Furthermore, consistent with the data from mice, transfection with the Sja-miR-71a mimic also down-regulated TGF-β-mediated IL-13Rα1, p-JAK1, and p-SATA6 expression (Figure 6(e,f)). Thus, we conclude that Sja-miR-71a suppresses TGF-β1/SMAD and IL-13/STAT6 pathways by downregulating Sema4D expression.

### Sja-miR-71a suppression of liver fibrosis is partly mediated by regulating Th1, Th2, Th17, and Treg balance via inhibiting Sema4D

**Figure 7.**
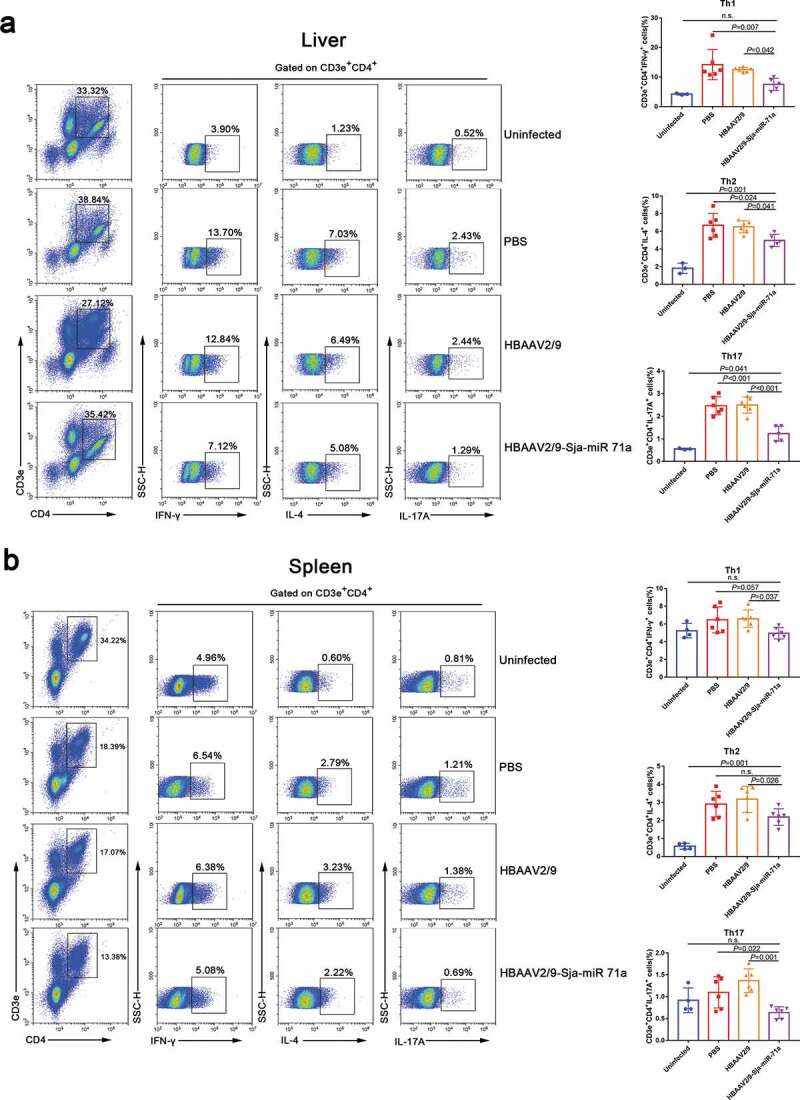
Sja-miR-71a decreased Th1, Th2 and Th17 percentages in the livers and spleens of *S. japonicum*-infected mice. (a). The percentage of Th1 (CD3e^+^CD4^+^IFN-γ^+^), Th2 (CD3e^+^CD4^+^IL-4^+^) and Th17 (CD3e^+^CD4^+^IL-17A^+^) intrahepatic lymphocytes were analysed by FACS (left), and the results of the statistical analysis are shown (right) (*n* = 3–6 per group). (b). The percentage of Th1, Th2 and Th17 cells in the spleens were analysed by FACS (left), and the results of the statistical analysis are shown (right) (*n* = 4–6 per group). Results are shown as mean ± SD (one-way ANOVA Dunnett’s multiple comparison test).

**Figure 8.**
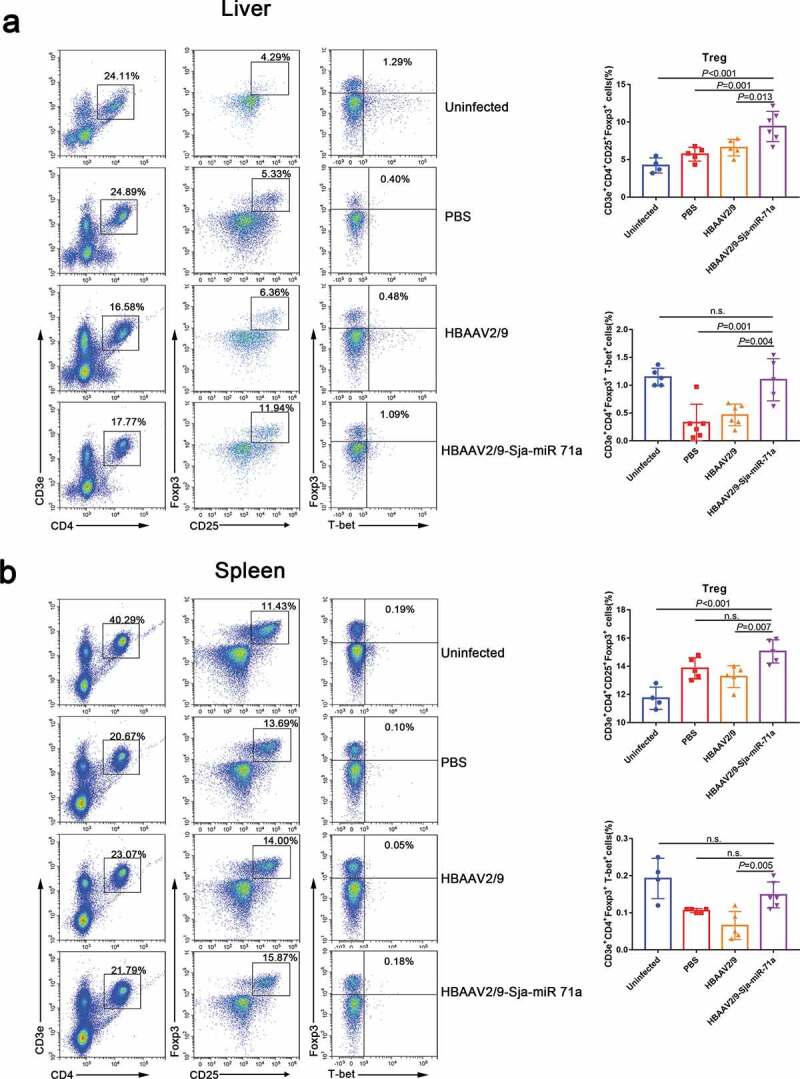
Sja-miR-71a increased Treg and T-bet^+^Treg percentages in the livers and spleens of *S. japonicum*-infected mice. (a). Treg (CD3e^+^CD4^+^CD25^+^Foxp3^+^) and T-bet+Treg (CD3e^+^CD4^+^ Foxp3^+^T-bet^+^) percentages of intrahepatic lymphocytes were analysed by FACS (left), and the results of the statistical analysis are shown (right) (*n* = 4–5 per group). (b). Treg and T-bet+Treg percentages of the spleens were analysed by FACS (left), and the results of the statistical analysis are shown (right) (*n* = 4–5 per group). Results are shown as mean ± SD (one-way ANOVA Dunnett’s multiple comparison test).

Sema4D is also expressed by T cells and is crucially involved in T-cell activation [35], and we found there has a group of CD3e^+^CD4^+^ cells around the granuloma of *S. japonicum* (Supplementary Figure 7). Therefore, we assessed the changes in Th1, Th2, Th17 and Treg subset levels in livers and spleens by flow cytometry. We observed that the percentages of CD3e^+^CD4^+^IFN-γ^+^ (Th1), CD3e^+^CD4^+^IL-4^+^ (Th2) and CD3e^+^CD4^+^IL-17A^+^ (Th17) cells in the livers of HBAAV2/9-Sja-miR-71a-treated *S. japonicum*-infected mice were significantly lower (*P* < 0.05) than those in the livers of *S. japonicum*-infection control groups (Figure 7(a,b)). We then showed that the percentages of CD3e^+^CD4^+^CD25^+^Foxp3^+^ cells in both livers and spleens of HBAAV2/9-Sja-miR-71a-treated *S. japonicum*-infected mice were significantly higher (*P* < 0.05) than those in the *S. japonicum*-infection control groups (Figure 8(a,b)). T-bet acts as an important transcription factor and controls Treg cell migration, homoeostasis, and function during type-1 inflammatory responses [36]. Interestingly, we observed that the percentages of CD3e^+^CD4^+^ Foxp3^+^T-bet^+^ (T-bet^+^Treg) in livers and spleens of HBAAV2/9-Sja-miR-71a-treated *S. japonicum*-infected mice were higher (*P* < 0.05) than those in *S. japonicum*-infection control groups (Figure 8(a,b)).

Th2 cytokines are associated with persistent liver fibrosis in human *S. japonicum* infection [37], and Th17 can promote the proliferation and activation of stellate cells [38]. However, Treg cells can inhibit T-cell proliferation and T-effector function [39]. Thus, we concluded that Sja-miR-71a suppression of liver fibrosis is partly mediated by regulating the balance of Th1, Th2, Th17 and Treg cells via inhibiting Sema4D.

## Discussion

**Figure 9.**
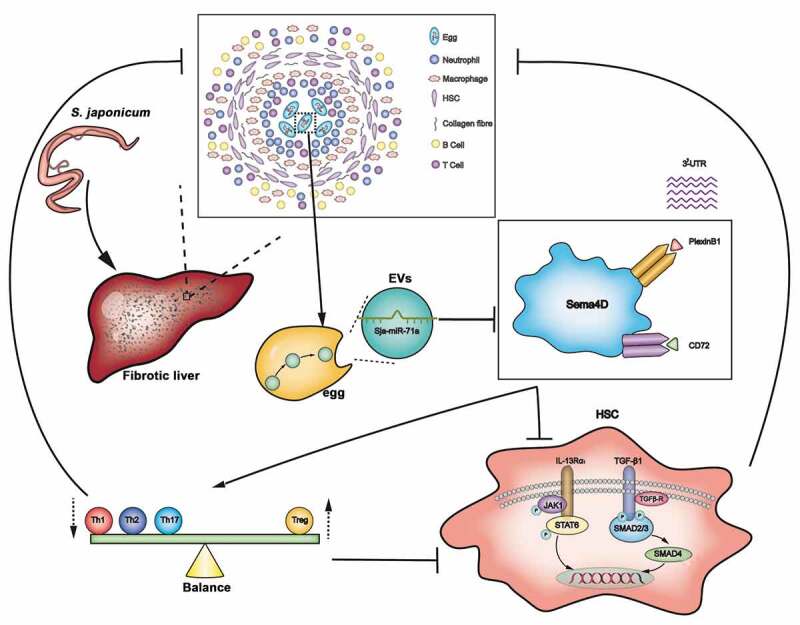
Model: Sja-miR-71a in *S. japonicum* egg-derived EVs suppresses liver fibrosis in schistosomiasis by targeting semaphorin 4D. *S. japonicum* egg-derived EVs can inhibit the activation of HSCs, and this inhibition is mediated via the high expression of Sja-miR-71a. Sja-miR-71a attenuates the liver fibrosis in *S. japonicum* infection by Sema4D/TGF-β1 axis and Sema4D/IL-13 axis. In addition, Sja-miR-71a suppression of liver fibrosis is partly mediated by regulating Th1, Th2, Th17 and Treg balance via inhibiting Sema4D.

The present study provides evidence with a functional role of Sja-miR-71a in *S. japonicum* egg-derived EVs in schistosomiasis-induced liver fibrosis. We showed that *S. japonicum* egg-derived EVs can inhibit the activation of HSCs, and this inhibition is mediated via the high expression of Sja-miR-71a. Sja-miR-71a attenuates the pathological progression of liver fibrosis in *S. japonicum* infection. Sja-miR-71a suppresses liver fibrosis by Sema4D/TGF-β1 axis and Sema4D/IL-13 axis. In addition, Sja-miR-71a suppression of liver fibrosis is partly mediated by regulating Th1, Th2, Th17 and Treg balance via inhibiting Sema4D (Figure 9).

In *Schistosoma*-host interactions, the host immune system works towards binding and accelerating the death of *S. japonicum* eggs by fibrotic reactions; accordingly, the *S. japonicum* eggs have evolved several ways to inhibit host fibrosis. Recent studies have shown that *S. japonicum* eggs and soluble egg antigens suppress the activation of HSCs to limit the progression of liver fibrosis [2,4–6,40]. EVs secreted from various parasites play important roles in host–parasite communication [41]. We isolated *S. japonicum* egg EVs by ultracentrifugation and found that they can inhibit the activation of HSCs. However, the results of the study by Xing He et al. showed that *schistosome* egg-derived EVs promoted activation of HSCs [42]. We thought the possible reason is that Xing He et al. used EVs isolated from the Exosome Precipitation Kit instead of ultracentrifugation, and studies showed that commercially available kit not much thought was given to potential contamination of moderate-sized vesicles and chemical impurities from the precipitant could also be the cause of toxicity [43,44]. EVs transmission of miRNAs and crosstalk with the downstream consequences ultimately affect disease progression [45]. To better understand the effects of *S. japonicum* egg-derived EVs in HSC inhibition, we sequenced and analysed the miRNAs of *S. japonicum* egg-derived EVs. We found that Sja-miR-71a is the most highly expressed miRNA.

Samoil *et al*. and Nowacki *et al*. showed *S. mansoni* EVs contain sma-miR-71a [17,46], and sma-miR-71a can be detected in sera of infected mice [46]. Fromm *et al*. showed fhe–miR-71a was expressed in *Fasciola hepatica* EVs [47]. A previous study indicated that Sja-miR-71 was related to sexual development of *S. japonicum* [48]. However, to our best knowledge, there were no reports about Sja-miR-71a in fibrosis. Interestingly, we found that Sja-miR-71a significantly inhibited the activation of HSCs *in vitro*. In addition, our findings demonstrate that Sja-miR-71a attenuates the pathological progression of and liver fibrosis in *S. japonicum* infection *in vivo*.

To further evaluate the molecular mechanisms of Sja-miR-71a in liver fibrosis, we aimed to identify direct target genes that might be controlled by Sja-miR-71a and identified Sema4D as a direct target of Sja-miR-71a. We found Sema4D in serum of schistosomiasis japonicum patients were increased. Interestingly, Sema4D expression was down-regulated *in vitro* and *in vivo* upon Sja-miR-71a overexpression. Sema4D binds with two receptors – Plexin B1 and CD72 [31]. In line with decreased Sema4D expression, both Plexin B1 and CD72 were down-regulated after Sja-miR-71a treatment. To the best of our knowledge, there are no reports yet on whether Sema4D and Sema4D receptors (Plexin B1 and CD72) are expressed in HSCs. In this study, we demonstrated that activated HSCs highly express Sema4D and Sema4D receptors (Plexin B1 and CD72), which can be downregulated by Sja-miR-71a. In addition, inhibiting the expression of Sema4D, Plexin B1 and CD72 could suppress HSC activation.

Studies have shown that Sema4D can directly increase TGF-β1; IL-13 and TGF-β1 were decreased in a Sema4D^−/−^ mouse model of experimental asthma [31,32]. In addition, both TGF-β1/SMAD and IL-13/STAT6 pathways have been identified as major pathways that promote liver fibrosis in schistosomiasis by activating HSCs [33,34,49]. Therefore, we speculated that Sja-miR-71a suppresses liver fibrosis through the Sema4D/TGF-β1 and Sema4D/IL-13 axes. Abundant evidence shows that SMADs are critical for TGF-β family signalling [50]. p-SMAD 2/3 and SMAD4 act as pivotal downstream effectors of TGF-β1 to convey signals from TGF-β receptors to the nucleus. TGF-β activates HSCs by transmitting their signals through the c-Jun N-terminal kinase-dependent SMAD2/3 phosphorylation [51]. SMAD4, which is the downstream protein of p-SMAD 2/3 in the TGF-β1/SMAD pathway, promotes fibrosis [52]. We found that the expression of TGF-β1, p-SMAD 2/3, and SMAD4 was up-regulated in the schistosome-infected livers and decreased after HBAAV2/9-Sja-miR-71a treatment, and p-SMAD 2/3 and SMAD4 were down-regulated by Sja-miR-71a treatment *in vitro*. IL-13 induces TGF-β1 production, a potent regulator of extracellular matrix formation [53]. IL-13 activates JAK1 in the cytoplasm mediated by IL-13Rα1/IL-4Rα receptor systems [54]. JAK1 was induced to phosphorylation, thereby activating the JAK/STAT6 signalling pathway; then, p-SATA6 forms a homologous dimer that enters the nucleus and binds to the promoter of the fibrogenic gene and initiates its transcription. We observed that IL-13Rα1, p-JAK1 and p-SATA6 were down-regulated by Sja-miR-71a treatment *in vivo* and *in vitro*. Thus, we conclude that Sja-miR-71a suppresses liver fibrosis by inhibiting the Sema4D/TGF-β1 and Sema4D/IL-13 axes.

In the immune system, Sema4D is constitutively expressed on T cells and is crucially involved in T-cell activation [55]. We found there has a group of CD3e^+^CD4^+^ cells around the granuloma of *S. japonicum*. Therefore, we assessed the changes in Th1, Th2, Th17 and Treg subset levels in livers and spleens. Our results showed that Th1, Th2, and Th17 subset levels in the livers and spleens of HBAAV2/9-Sja-miR-71a-treated *S. japonicum*-infected mice were lower than control groups. Treg subset levels in both livers and spleens of HBAAV2/9-Sja-miR-71a-treated *S. japonicum*-infected mice were increased compared with the levels in *S. japonicum*-infection control groups. A previous study showed that T cell proliferation was downregulated in OVA323–339-restimulated Sema4D^−/−^ cell cultures, and Treg numbers were increased in the spleens of Sema4D^−/−^ mice [32], which is consistent with our data.

Treg cells regulate the balance of Th1/Th2 responses in the process of *S. japonicum* infection [56]. Th17 has been linked with severe hepatic inflammation in schistosomiasis [57]. In addition, fibrosis is state driven by Th responses, Th2 cytokines are associated with persistent liver fibrosis in human *S. japonicum* infection [37,58], and Th17 can promote the proliferation and activation of stellate cells [38]. However, Treg cells can inhibit T-cell proliferation and T-effector function [39]. T-bet acts as an important transcription factor and controls Treg cell migration, homoeostasis, and function [36]. Interestingly, we observed that the percentage of T-bet^+^Treg cells in the livers and spleens of HBAAV2/9-Sja-miR-71a-treated *S. japonicum*-infected mice were increased. Thus, we concluded that Sja-miR-71a-based suppression of liver fibrosis is partly mediated by regulating the Th1/Th2/Th17/Treg balance by inhibition of Sema4D.

In summary, our data show that Sja-miR-71a in *S. japonicum* egg-derived EVs suppresses liver fibrosis in schistosomiasis by directly targeting Sema4D. We believe these results contribute to further understanding the molecular mechanisms underlying *Schistosoma*–host interactions, and Sema4D may be a potential target for schistosomiasis liver fibrosis treatment.

## Supplementary Material

Supplemental Material
