# Supplementary material for: Sja-miR-71a in Schistosome egg-derived extracellular vesicles suppresses liver fibrosis caused by schistosomiasis via targeting semaphorin 4D
Source: J Extracell Vesicles. 2020 Jul 9;9(1):1785738. doi: 10.1080/20013078.2020.1785738 (PMC7480424; doi:10.1080/20013078.2020.1785738)
Supplement: Supplemental Material [file ZJEV_A_1785738_SM5583.docx]

**Supplementary Information**

**Supplementary materials and methods**

***EVs purification and identification***

To prepare *S. japonicum* EVs, *S. japonicum* eggs and worms were collected from infected mice at 45 days post-infection. *S. japonicum* eggs in liver were collected by Percoll with a density of 1.070. The eggs and worms were maintained in RPMI-1640 culture medium, the culture supernatant was collected for further experiments.

EVs in eggs were analyzed by transmission electron microscopy. The eggs were fixed, washed, dehydrated, and embedded in araldite. Then, ultrathin sections were cut and contrasted with 1% methanolic uranyl acetate and Reynold’s solution of lead citrate. The sections were observed under a Hitachi H-300 transmission electron microscope. EVs were purified by differential centrifugation and following validation by electron microscopy and Nanoparticle Tracking Analyses (NTA). Briefly, the eggs and worms culture supernatants and human serum were centrifuged at a low speed (700 g for 30 min at 4°C) (15ml polypropylene tube, swinging bucket rotor, model A-4-44, 5804R Refrigerated Centrifuge, Eppendorf, Germany), and the resulting supernatants were centrifuged at 3500 g for 30 min at 4°C (15ml polypropylene tube, swinging bucket rotor, model A-4-44, 5804R Refrigerated Centrifuge, Eppendorf, Germany). The supernatants were transferred into 1.5 ml polypropylene tube (Eppendorf, Germany) with a micropipette, and then centrifuged at 20,000 g for 60 min at 4°C (Fixed angle rotor, angle is 45 degrees, model #3331, D-37520 Refrigerated Centrifuge, Thermo Electron Corporation, USA); the resulting supernatants were transferred into Quick-Seal Centrifuge tube (Beckman Coulter, USA) and centrifuged at 120,000 g for 90 min at 4°C in an Optima L-100xp tabletop ultracentrifuge (Swinging bucket rotor, model SW40 Ti, Optima L-100xp, Beckman Coulter, USA). The resultant pellet (EVs) was diluted with phosphate-buffered saline (PBS). Further, negative-staining transmission electron microscopy (TEM) was used to analyze the EVs. EVs were loaded on a copper grid and negatively stained with 3% (w/v) aqueous phosphotungstic acid for 1 min. The grid was then examined using a FEI Tecnai G2 Sprit Twin TEM (FEI, USA). In addition, EVs particles were analyzed using NTA (NanoSight NS300, Malvern Instruments, United Kingdom). We used the NanoSight NS300 instrument equipped with a sCMOS camera, 488 nm laser (Blue), NTA 3.3 Dev Build 3.3.301 software, and the number of frames was 749.

***Cell culture and treatment***

For EVs and Recombinant Human Semaphorin 4D (Sema4D) (Novoprotein, China) treatment, LX2 cells were exposed to recombinant TGF-β1 (PeproTech, USA) or EVs. For microRNA mimic and siRNA treatment, cells were incubated in serum-free medium for starvation overnight. Cells were then stimulated with microRNA mimic and siRNA using RNAiMAX (Invitrogen, USA).

SiRNAs for transfection

| **siRNA (gene)** | **Sequence** | |
| --- | --- | --- |
|  | **sense (5’-3’)** | **Antisense (5’-3’)** |
| HS-Sema4D | GGAUGGGACUGUCUAUGAUdTdT | AUCAUAGACAGUCCCAUCCdTdT |
| HS-PlexinB1 | CGAGUAUGAUGUCAAUUCUdTdT | AGAAUUGACAUCAUACUCGdTdT |
| HS-CD72 | GGGACAAAGCAGCGGUCAAdTdT | UUGACCGCUGCUUUGUCCCdTdT |

***RNA extraction and*** ***quantitative reverse-transcription PCR (qRT-PCR)***

Total RNA was harvested using TRIzol according to manufacturer’s instructions. The expression of target mRNA and microRNA was determined using the SYBR Green Master Mix kit (Takara, Japan). GAPDH, β-actin or U6 snRNA were used as an internal control, and the fold change was calculated by the 2^-ΔΔCT^ method.

Quantitative Real Time PCR Primers’ sequences

| Gene | Forward (5’-3’) | Reverse (5’-3’) |
| --- | --- | --- |
| α-SMA (human) | CCAGGGCTGTTTTCCCATCC | GCTCTGTGCTTCGTCACCCA |
| Collagen I (human) | TGATGGGATTCCCTGGACCT | GGGCCTTGTTCACCTCTCTC |
| Sema4D (human) | ACTCACAGAGGATCGACGAC | TCTCCATCTGCGTCTGAGTC |
| β-actin (human) | CTGGCACCACACCTTCTACAATG | AATGTCACGCACGATTTCCCGC |
| α-SMA (Mouse) | CACAGCCCTGGTGTGCGACAAT | TTGCTCTGGGCTTCATCCCCCA |
| Collagen I (Mouse) | TCCTGCGCCTAATGTCCACCGA | AAGCGACTGTTGCCTTCGCCTC |
| Sema4D (Mouse) | CTGCTACAAGGGCTACCTGC | GCTCGACCAGTGTCTCCTTC |
| Efna5 (Mouse) | AACCAGCAGATGACACCGTA | ATCGCCAGGAGGAACAGTAG |
| Rnf115 (Mouse) | CGGACAGACAATAGCACAGC | TCTTGGTCCAGTGGATTGCT |
| Mrgprh (Mouse) | ACATCCTCCATCTCGCCATT | AAGAGGATGGCGTAGCTCTC |
| Pex7 (Mouse) | GGGATGTGAAGACCACAGGA | TACACCAGTCACAGCTCAGG |
| Olfr918 (Mouse) | CATGCTGACTGGAAATGGCT | AGGATTGAGGCCAATCAGGA |
| Slc13a5 (Mouse) | TGCCAGGTGTGCCTATGTTA | ACAATGAGACTGCCCAGGAA |
| Nr3c2 (Mouse) | TCAGACCTTGGAGCGTTCTT | GCAGCTGACGTTGACAATCT |
| Rab11b (Mouse) | TACCATCGGAGTGGAGTTCG | CTGAGCCTTGATGGTCTTGC |
| GAPDH (Mouse) | ACTCCACTCACGGCAAATTC | TCTCCATGGTGGTGAAGACA |
| Sja-miR-71a (Sj) | TGAAAGACGATGGTAGTGAGA | mRQ 3' Primer (Takara, Kyoto, Japan) |
| U6 | Takara, Kyoto, Japan | Takara, Kyoto, Japan |

***Small RNA sequencing and analysis***

Total RNA was isolated from EVs and subjected to quantitative and qualitative analyses to ensure the use of qualified samples for sequencing. A total of 2.5 ng RNA per sample was used as the input material for generation of the small RNA library. Following cluster generation, the library preparations were sequenced on an Illumina Hiseq 2500 platform (Illumina, USA) and paired-end reads were generated. After sequencing, the data were subjected to the following preliminary analyses, which were carried out by the Biomarker Corporation: quality control analysis, comparative analysis, target gene functional annotation, quantification of miRNA expression levels, mRNA differential gene expression analysis, and GO and KEGG enrichment analysis.

***Western blotting***

Liver tissues and LX2 cells were homogenized with RIPA lysis buffer in the presence of freshly added protease and phosphatase inhibitors (Thermo Fisher Scientific, USA). Lysates were then quantified and equal amounts of protein were subjected to 10% sodium dodecyl-polyacrylamide gel electrophoresis and transferred to a polyvinylidene fluoride (PVDF) blotting membrane (GE Healthcare Life Sciences, UK). The membranes were immunoblotted with the following antibodies: α-SMA, Collagen I, Sema4D, Plexin B1, CD72, IL-13Rα1, p-JKA1, p-STAT6, TGF-β1, p-SMAD2/3, and SMAD4; GAPDH antibody was used as an internal standard**.**

Antibodies List (Western blotting)

| Primary antibody | Working conditions | Catalogue No |
| --- | --- | --- |
| α-SMA | 1:1000 | Abcam ab32575 (Cambridge, UK) |
| Collagen I | 1:2000 | Proteintech 14695-1-AP (Wuhan, china) |
| Sema4D | 1:2000 | SAB 35396 (MD, USA) |
| PlexinB1 | 1:300 | Proteintech 23795-1-AP (Wuhan, china) |
| CD72 | 1:300 | Proteintech 13469-1-AP (Wuhan, china) |
| TGF-β1 | 1:600 | Proteintech 21898-1-AP (Wuhan, china) |
| p-SMAD2/3 | 1:500 | SAB 12241(MD, USA) |
| SMAD4 | 1:600 | Proteintech 10231-1-AP (Wuhan, china) |
| IL-13Rα1 | 1:1000 | Abcam ab79277 (Cambridge, UK) |
| p-JAK1 | 1:500 | SAB 13119 (MD, USA) |
| P-STAT6 | 1:1000 | Abcam ab54461 (Wuhan, china) |
| GAPDH | 1:5000 | Sigma-Aldrich G9295 (Missouri, USA) |

***Immunohistochemistry and immunoflourescence analysis***

Liver tissues were fixed in 4% neutral buffered formalin and embedded in paraffin. Sections were dewaxed and incubated with GFAP, α-SMA, Sema4D, Plexin B1, CD72, IL-13Rα1, p-JKA1, p-STAT6, TGF-β1, p-SMAD2/3, SMAD4, CD3e and CD4 antibodies overnight at 4℃. The sections were then incubated with the indicated secondary antibodies. The sum of the IOD was analyzed by Image-Pro Plus 6.0.

Antibodies List (Immunohistochemistry and immunoflourescence analysis)

| Primary antibody | Working conditions | Catalogue no. |
| --- | --- | --- |
| GFAP | 1:100 | Proteintech 60190-1-AP (Wuhan, china) |
| α-SMA | 1:100 | Abcam ab7817 (Cambridge, UK) |
| Sema4D | 1:100 | SAB 35396 (MD, USA) |
| PlexinB1 | 1:100 | Proteintech 23795-1-AP (Wuhan, china) |
| CD72 | 1:100 | Proteintech 13469-1-AP (Wuhan, china) |
| TGF-β1 | 1:100 | Proteintech 21898-1-AP (Wuhan, china) |
| p-SMAD2/3 | 1:100 | SAB 12241 (MD, USA) |
| SMAD4 | 1:100 | Proteintech 10231-1-AP (Wuhan, china) |
| IL-13Rα1 | 1:100 | Abcam ab79277 (Cambridge, UK) |
| p-JAK1 | 1:100 | SAB 13119 (MD, USA) |
| P-STAT6 | 1:100 | Abcam ab54461 (Cambridge, UK) |
| CD3e | 1:100 | Affinity biosciences DF6594 (OH, USA) |
| CD4 | 1:100 | Abcam ab25804 (Cambridge, UK) |

***Flow cytometry***

For spleens, single-cell suspensions were prepared. For livers, single cells were resuspended in 40% Percoll and centrifuged at 1,000 *g* for 15 min without braking. Intrahepatic lymphocytes in the pellet were collected. Th1, Th2, Th17, and Treg subsets were analyzed by flow cytometry. For Treg subsets, the cells were stained with CD3e, CD4, and CD25; the cells were then stained with Foxp3 and T-bet antibodies after fixation and permeabilization. For Th1, Th2, and Th17 subsets, cells were cultured with 50 ng/ml phorbol 12-myristate 13-acetate (PMA), 500 ng/mL ionomycin, and 10 μg/mL brefeldin A (Sigma-Aldrich, USA) for 6 h. Then, cells were washed and stained for CD3e and CD4. In addition, cells were stained with intracellular interferon-γ (IFN-γ), IL-4, and IL-17A antibodies. Images were acquired on a CytoFLEX S flow cytometer (Beckman Coulter, USA).

Antibodies List (Flow cytometry)

| Antibody | Order no. | Manufacturer |
| --- | --- | --- |
| CD3e | 557596 | BD Biosciences, San Diego, CA, USA |
| CD4 | 553052 | BD Biosciences, San Diego, CA, USA |
| CD25 | 47-0257-41 | eBiosciences, San Diego, CA, USA |
| Foxp3 | 12-4776-41 | eBiosciences, San Diego, CA, USA |
| T-bet | 563318 | BD Biosciences, San Diego, CA, USA |
| IFN-γ | 557724 | BD Biosciences, San Diego, CA, USA |
| IL-4 | 554436 | BD Biosciences, San Diego, CA, USA |
| IL-17A | 563354 | BD Biosciences, San Diego, CA, USA |

**Supplementary Figures**

**
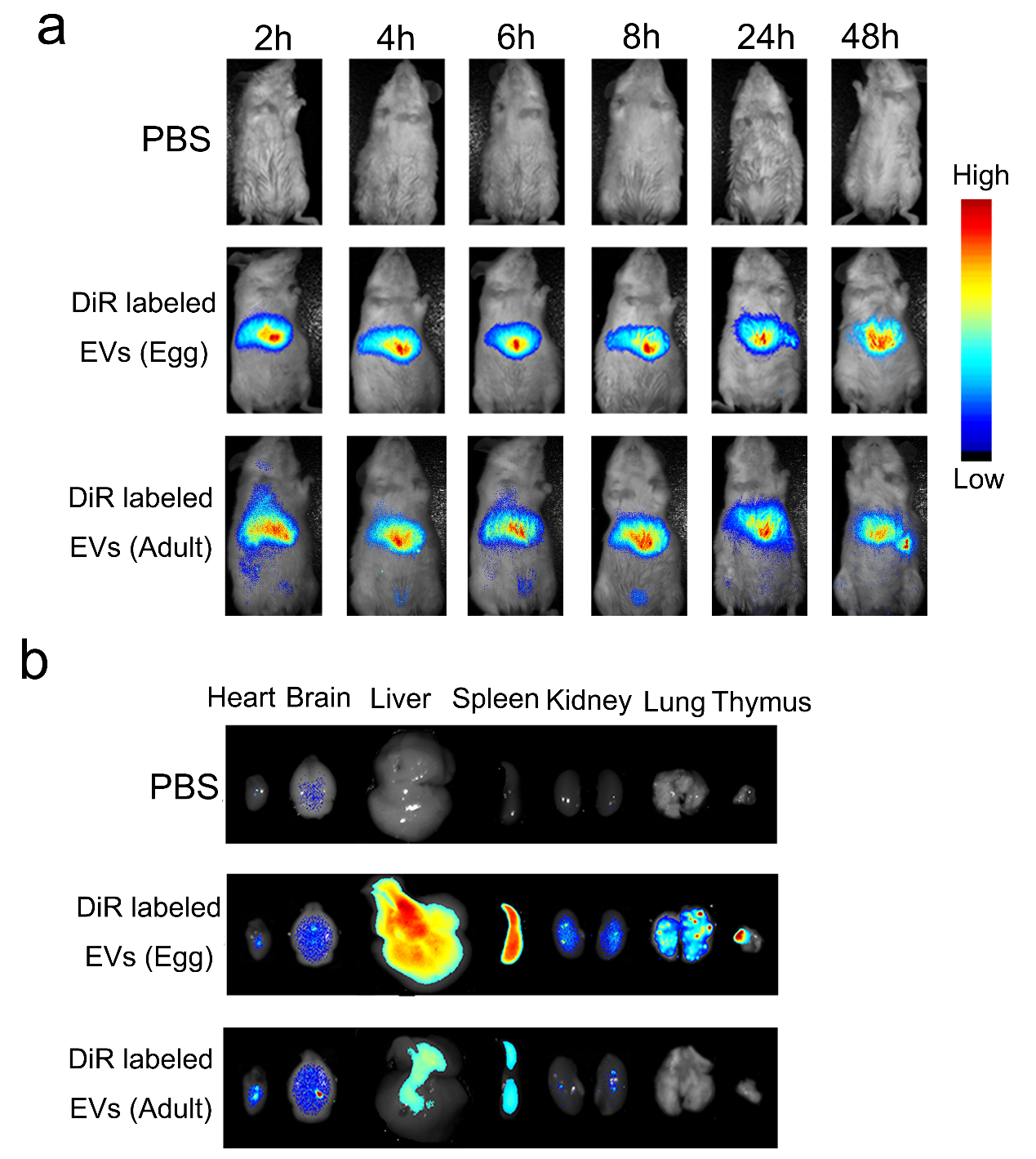
**

**Supplementary Fig****ure 1. a.** *S. japonicum* egg-derived EVs and *S. japonicum* adult-derived EVs were stained with DiR (1,1′‐dioctadecyl‐3,3,3′,3′‐tetramethylindotricarbocyanine iodide), and mice were injected via the tail vein with DiR-labeled EVs. In vivo real-time imaging of the distribution of DiR labeled EVs at 2, 4, 6, 8, 24, and 48 h. **b.** At the end of the last total body scan, 48 h post-injection, mice were euthanized, organs were removed for *ex vivo* imaging, and relative quantitative representation of fluorescent signals was calculated.


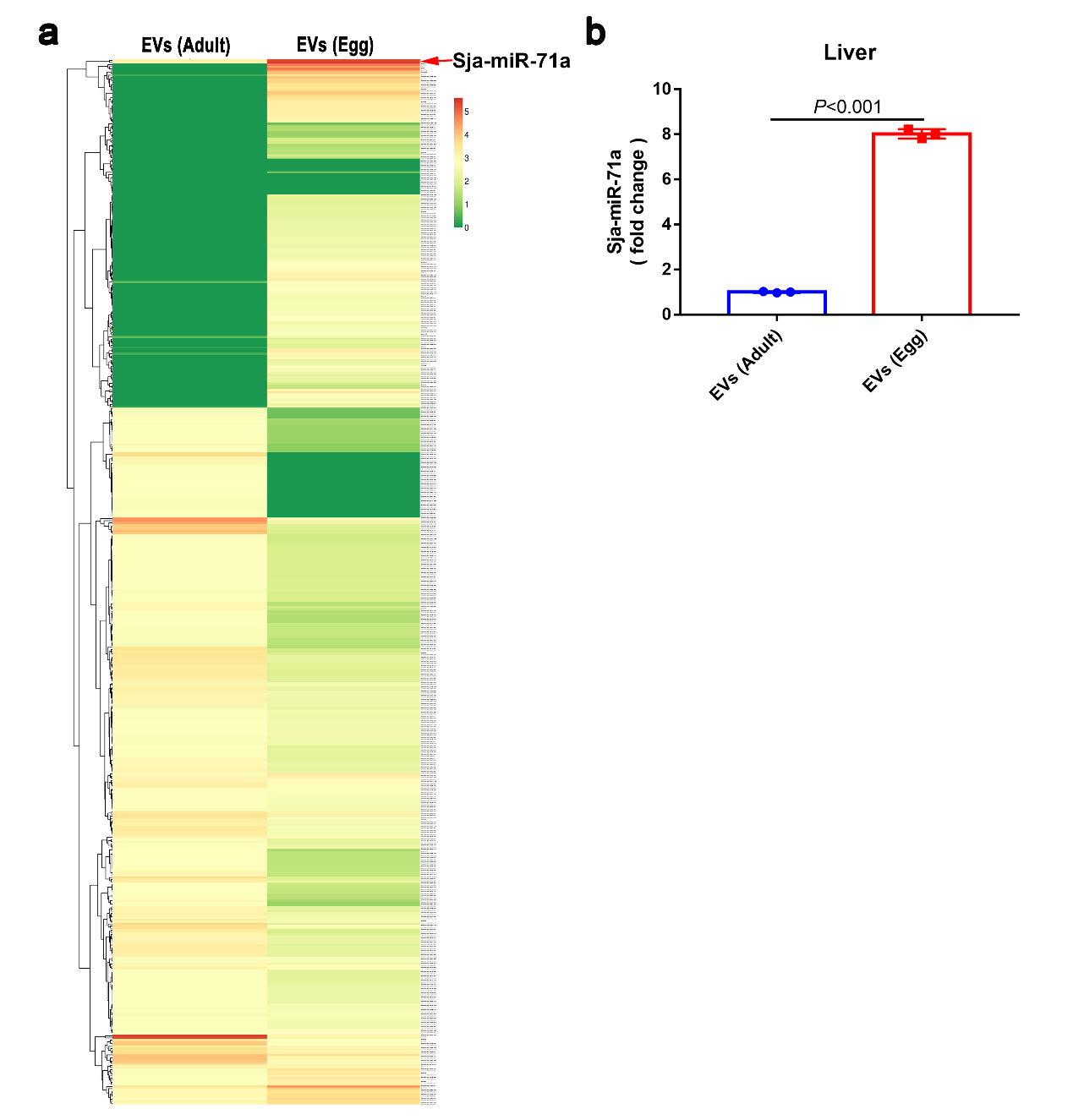


**Supplementary Figure 2. a.** miRNAs of *S. japonicum* egg-derived EVs and *S. japonicum* adult-derived EVs were sequenced and analyzed. The heat map analysis shows differential expression of miRNAs, and Sja-miR-71a is the most highly expressed miRNA in *S. japonicum* egg-derived EVs. Bright green = low expression; bright red = high expression. **b.** Mice were injected via the tail vein with *S. japonicum* egg-derived EVs and *S. japonicum* adult-derived EVs. 24 h post-injection, mice were euthanized, Sja-miR-71a in liver was analyzed by quantitative reverse transcription PCR (qRT-PCR). Results are shown as mean ± SD. Unpaired two-sample *t*-test.

**
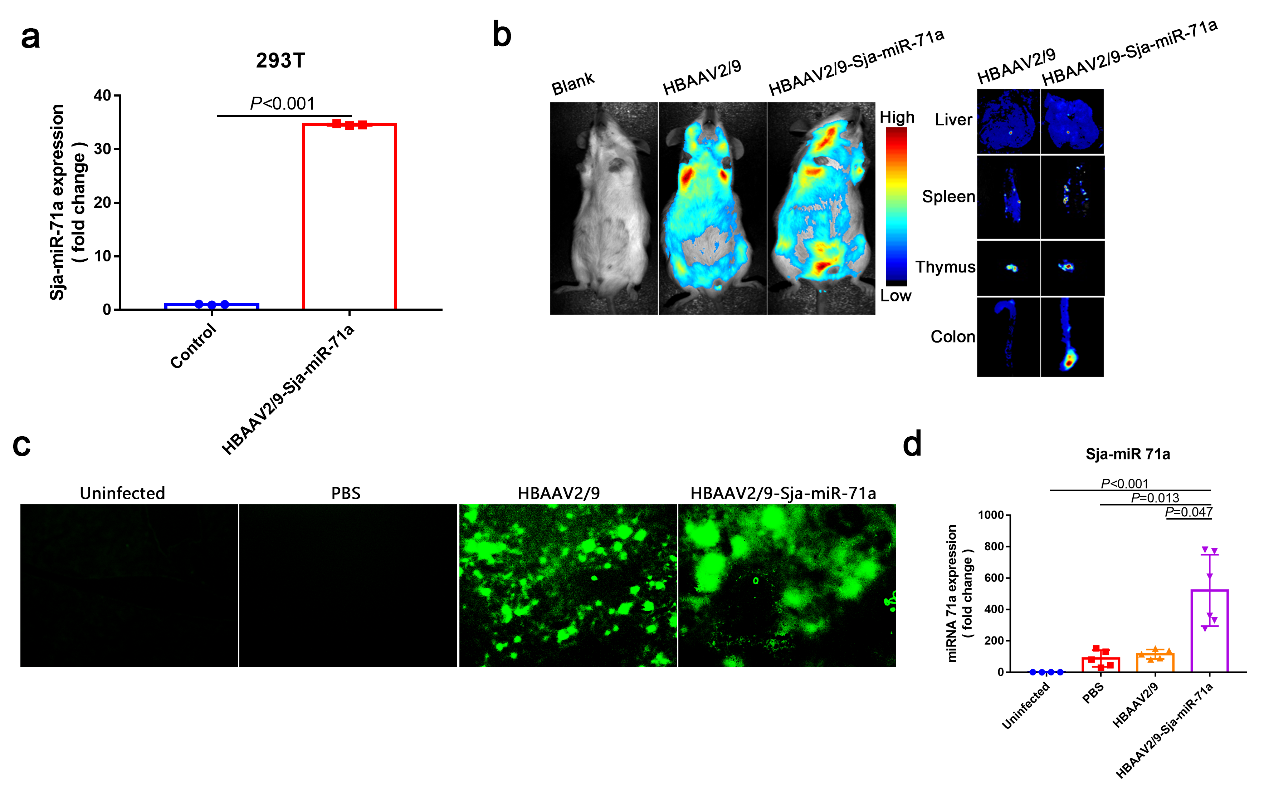
**

**Supplementary Figure 3. HBAAV2/9-Sja-miR-71a was constructed and the mice were successfully transfected (HBAAV2/9 can express GFP protein).** **a.** HBAAV2/9-Sja-miR-71a was constructed and the expression level of Sja-miR-71a was analyzed by qRT-PCR. **b.** In vivo imaging of the distribution of HBAAV2/9-Sja-miR-71a in mice; HBAAV2/9-Sja-miR-71a shows colonization in the liver, spleen, thymus, and colon. **c.** HBAAV2/9-Sja-miR-71a colonization in the liver was analyzed by fluorescence microscopy. **d.** Expression level of Sja-miR-71a in mice livers was analyzed by qRT-PCR (n=4–6 per group). Results are shown as mean ± SD. **a**: Unpaired two-sample *t*-test. **d**: One-way ANOVA Dunnett’s multiple comparison test.


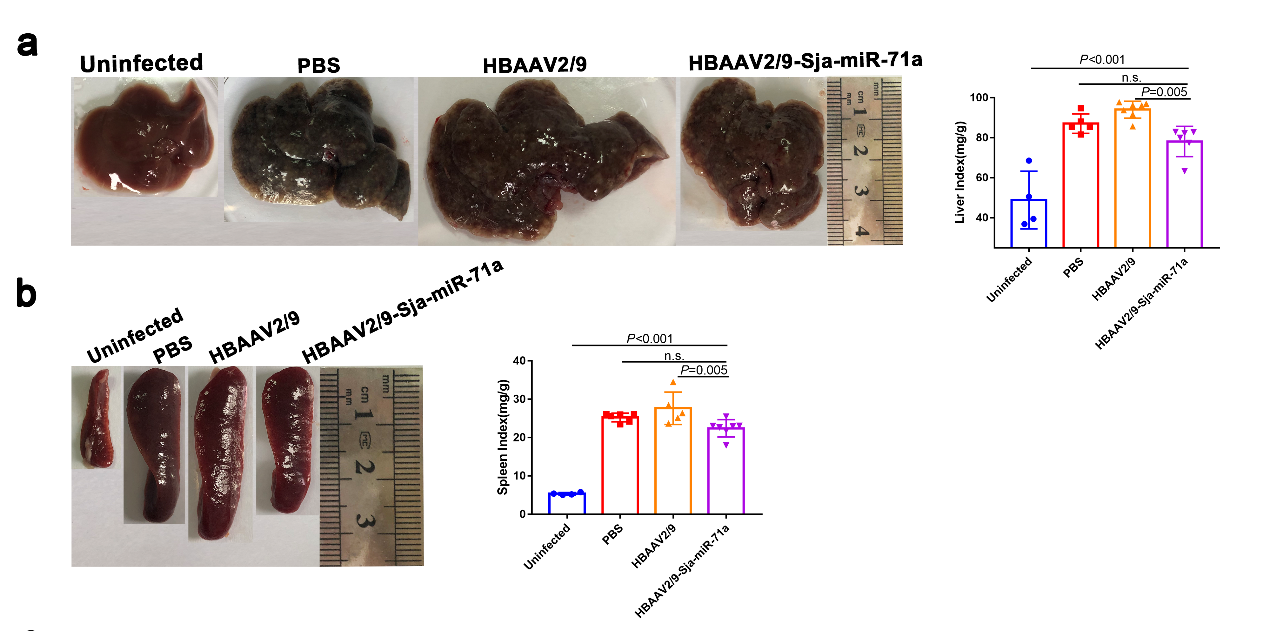


**Supplementary Figure 4. HBAAV2/9-Sja-miR-71a attenuates the pathological progression of *S. japonicum* infection. a.** The macroscopic appearance of livers and liver index (n=4–7 per group). **b.** The macroscopic appearance of spleens and spleen index (n=4–7 per group). Results are shown as mean ± SD (one-way ANOVA Dunnett’s multiple comparison test).


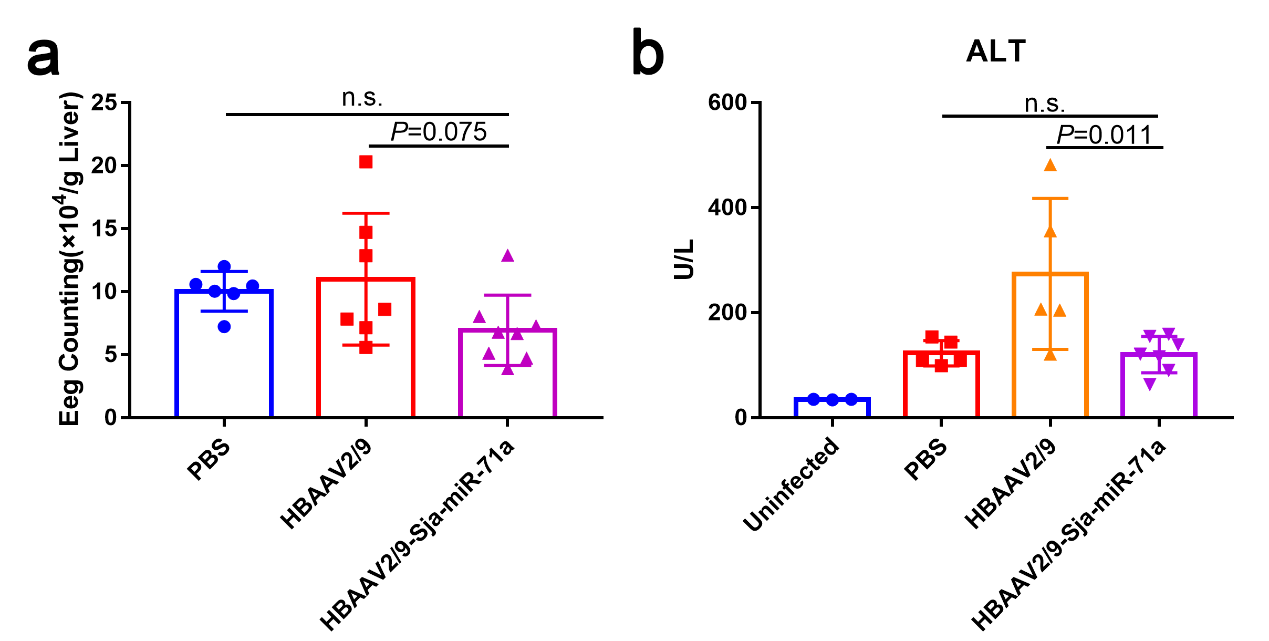


**Supplementary Figure 5. Liver egg burdens were counted and serum ALT levels were detected.** **a.** Liver egg burdens were counted after the liver tissue was digested by 4% KOH (n=6–7 per group). **b.** Serum ALT levels were detected (n=3–6 per group). (Results are shown as mean ± SD, one-way ANOVA Dunnett’s multiple comparison test).


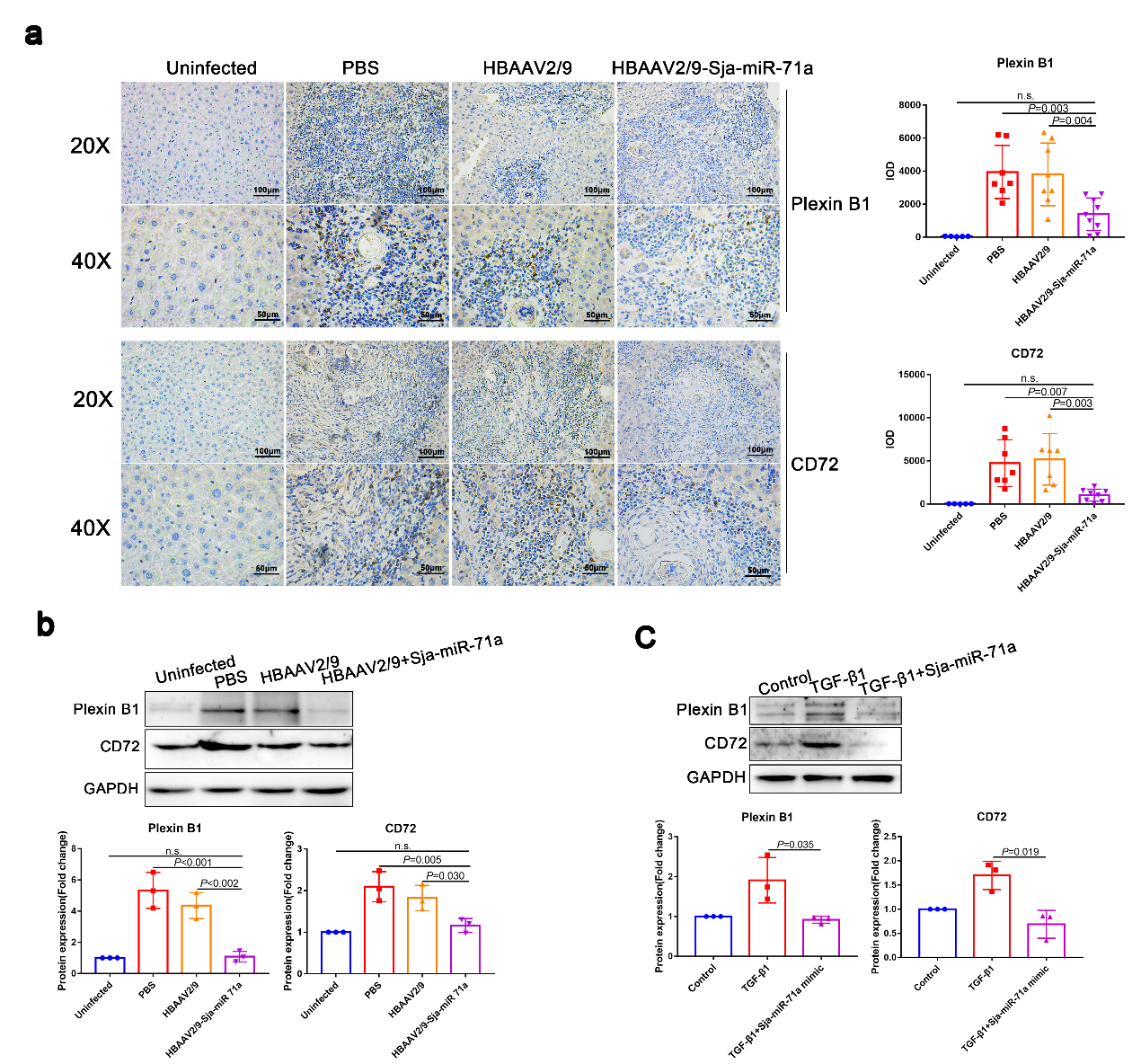


**Supplementary Figure 6.** **Expression of Plexin B1 and CD72 were down-regulated by Sja-miR-71a.** **a, b.** Expression of Plexin B1 and CD72 in mice livers were analyzed by immunohistochemistry (the sum of the IOD was analyzed by Image-Pro Plus 6.0， n=5–8 per group) and western blotting. **c.** Expression levels of Plexin B1 and CD72 were determined by western blotting for LX2 cells treated with PBS, TGF-β1, and TGF-β1+Sja-miR-71a mimic. Results are shown as mean ± SD (one-way ANOVA test).


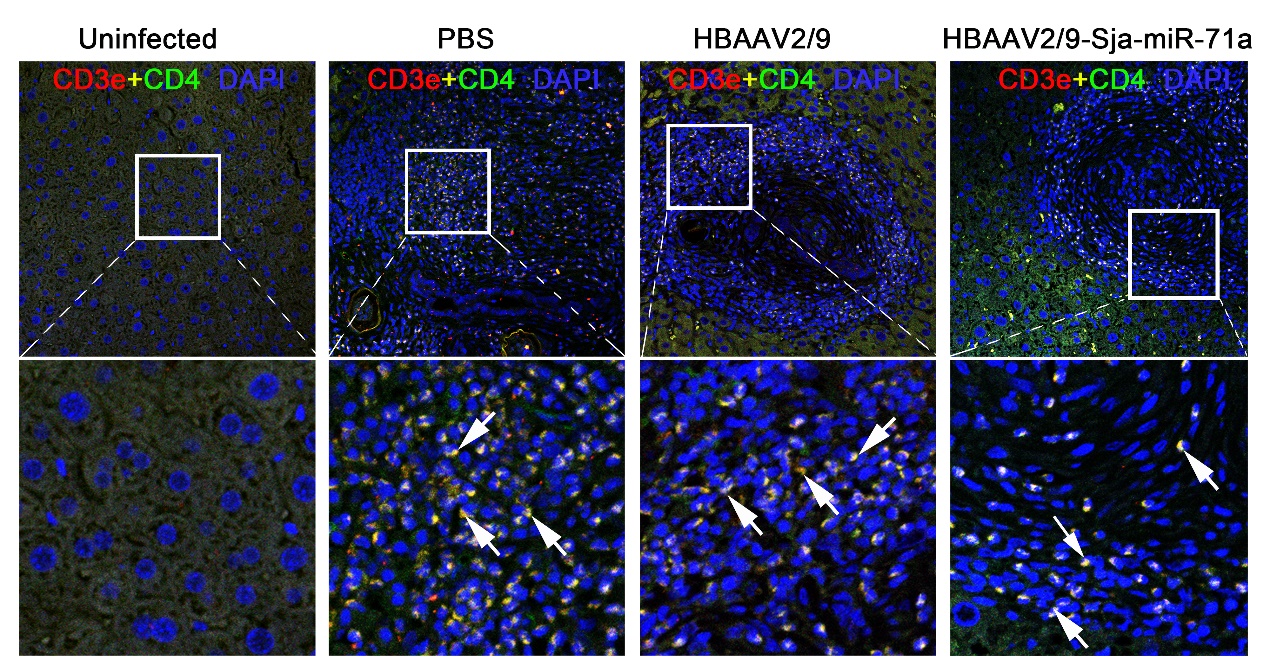


**Supplementary Figure 7.** The co-localization of CD3e and CD4 in liver sections was observed, DAPI was used as a counterstain.

**Supplementary Table 1.** Top 10 highly expressed miRNAs in *S. japonicum* egg-derived EVs.

| #ID | *S. japonicum* egg-derived EVs (TPM) | Adult *S. japonicum* adult-derived EVs (TPM) |
| --- | --- | --- |
| Sja-miR-71a | 269840.2488 | 987.6010092 |
| Sja-miR-36-3p | 208966.8011 | 1963.873262 |
| Sja-bantam | 68562.08804 | 0 |
| Sja-miR-71b-5p | 66885.87364 | 0 |
| Sja-miR-2162-3p | 32457.26221 | 2902.16086 |
| Sja-miR-1 | 25699.43168 | 0 |
| Sja-miR-190-5p | 19308.62777 | 0 |
| Sja-miR-7-5p | 10163.23226 | 0 |
| Sja-miR-3492 | 9296.746706 | 0 |
| unconservative_SJC  _S000045_5447 | 8687.558176 | 0 |
